# *In utero* and Lactational Exposure to Acetamiprid Induces Abnormalities in Socio-Sexual and Anxiety-Related Behaviors of Male Mice

**DOI:** 10.3389/fnins.2016.00228

**Published:** 2016-06-03

**Authors:** Kazuhiro Sano, Tomohiko Isobe, Jiaxin Yang, Tin-Tin Win-Shwe, Mitsuha Yoshikane, Shoji F. Nakayama, Takaharu Kawashima, Go Suzuki, Shunji Hashimoto, Keiko Nohara, Chiharu Tohyama, Fumihiko Maekawa

**Affiliations:** ^1^Center for Health and Environmental Risk Research, National Institute for Environmental StudiesTsukuba, Japan; ^2^Center for Environmental Biology and Ecosystem Studies, National Institute for Environmental StudiesTsukuba, Japan; ^3^Center for Material Cycles and Waste Management Research, National Institute for Environmental StudiesTsukuba, Japan; ^4^Center for Environmental Measurement and Analysis, National Institute for Environmental StudiesTsukuba, Japan; ^5^Faculty of Medicine, University of TsukubaTsukuba, Japan

**Keywords:** neonicotinoids, acetamiprid, *In utero* and lactational exposure, sociosexual behavior, anxiety-related behavior

## Abstract

Neonicotinoids, a widely used group of pesticides designed to selectively bind to insect nicotinic acetylcholine receptors, were considered relatively safe for mammalian species. However, they have been found to activate vertebrate nicotinic acetylcholine receptors and could be toxic to the mammalian brain. In the present study, we evaluated the developmental neurotoxicity of acetamiprid (ACE), one of the most widely used neonicotinoids, in C57BL/6J mice whose mothers were administered ACE via gavage at doses of either 0 mg/kg (control group), 1.0 mg/kg (low-dose group), or 10.0 mg/kg (high-dose group) from gestational day 6 to lactation day 21. The results of a battery of behavior tests for socio-sexual and anxiety-related behaviors, the numbers of vasopressin-immunoreactive cells in the paraventricular nucleus of the hypothalamus, and testosterone levels were used as endpoints. In addition, behavioral flexibility in mice was assessed in a group-housed environment using the IntelliCage, a fully automated mouse behavioral analysis system. In adult male mice exposed to ACE at both low and high doses, a significant reduction of anxiety level was found in the light-dark transition test. Males in the low-dose group also showed a significant increase in sexual and aggressive behaviors. In contrast, neither the anxiety levels nor the sexual behaviors of females were altered. No reductions in the testosterone level, the number of vasopressin-immunoreactive cells, or behavioral flexibility were detected in either sex. These results suggest the possibility that *in utero* and lactational ACE exposure interferes with the development of the neural circuits required for executing socio-sexual and anxiety-related behaviors in male mice specifically.

## Introduction

There is a growing concern that exposure to environmental chemicals in early life may interfere with brain development (Júlvez et al., 2016). In particular, neonicotinoid pesticides have drawn considerable attention. As a pesticide class, neonicotinoids are designed to overstimulate insect nicotinic acetylcholine receptors (nAChRs). These pesticides were previously thought to be relatively harmless to mammalian species because of their low binding affinities to mammalian nAChRs (Tomizawa and Casida, 1999, 2005). However, recent *in vivo* and *in vitro* studies have reported that neonicotinoids possess sufficient binding affinity and agonistic potential for mammalian nAChRs to exert nicotine-like effects that are stronger than originally believed (de Oliveira et al., 2010; Rodrigues et al., 2010; Li et al., 2011; Kimura-Kuroda et al., 2012). Neonicotinoids such as acetamiprid (ACE), imidacloprid, and clothianidin can bind to the α_4_ and β_2_ subunits of mammalian nAChRs (Tomizawa and Casida, 1999; Li et al., 2011; Kimura-Kuroda et al., 2012). The α_4_β_2_ nAChRs are present in various brain regions such as the amygdala, hypothalamus, substantia nigra, ventral tegmental area, raphe nuclei, hippocampus, and medial habenula (Cimino et al., 1995; Millar and Gotti, 2009), and regulate the development and functions of these regions (Dwyer et al., 2009; Takarada et al., 2012). These brain regions are involved in the regulation of socio-sexual behaviors, anxiety, depression, memory, and learning (Pfaff, 1989; Newman, 1999; Nelson and Trainor, 2007; Drevets et al., 2008; Gaskin and White, 2013; Russo and Nestler, 2013). Therefore, perinatal exposure to neonicotinoids is thought to impair specific behaviors by affecting the formation of neuronal circuits, including circuits involving these areas.

Since ACE has a higher affinity and potency for mammalian nAChRs compared to those of other neonicotinoids (Tomizawa and Casida, 1999; Kimura-Kuroda et al., 2012), we here studied effects of perinatal exposure to ACE on murine behaviors later in adulthood, focusing on socio-sexual and anxiety-related behaviors and behavioral flexibility. In addition to examining these adult behaviors, we evaluated the blood testosterone levels and the numbers of cells expressing arginine-vasopressin (AVP) in the hypothalamus because they are closely associated with socio-sexual and anxiety-related behaviors (Hull and Dominguez, 2007; Nelson and Trainor, 2007; Ho et al., 2010; Stevenson and Caldwell, 2012).

## Materials and methods

### Animals

Male and female C57BL/6J mice were purchased from CLEA Japan (Tokyo, Japan) and mated at the National Institute for Environmental Studies (NIES). Mice were housed in a room that was maintained at a constant temperature (24 ± 1°C) and humidity (50 ± 10%) with a 12/12-h light/dark cycle. Food and water were provided *ad libitum* unless otherwise specified. The presence of vaginal plugs was checked daily; gestational day (GD) 0 was defined as the day on which a vaginal plug was detected. The dams were administered ACE (Sigma-Aldrich, St. Louis, MO), dissolved in H_2_O, at doses of 0 mg/kg (control group), 1.0 mg/kg (low-dose group), or 10.0 mg/kg (high-dose group) by oral gavage from GD 6 to postnatal day (PND) 21. The litters were weaned from their mothers 2–3 h after the last ACE administration on PND 21 and housed with same-sex littermates. All procedures were approved by the Animal Care and Use Committee at the NIES and were conducted strictly in accordance with the NIES guidelines.

### Body weights

The body weight (BW) was measured at birth, at weaning (PND 21), and at 23–26 weeks of age, and the average body weight within a litter (BW/litter) was compared between treatment groups.

### Brain weights

At the time of weaning (PND 21), randomly selected mice that would not be used for behavioral testing were deeply anesthetized with isoflurane and decapitated. The brains were rapidly removed and weighed.

### ACE analysis in the brain

An ACE standard was obtained from Sigma-Aldrich. Acetamiprid-d3 (Fluka, Sigma-Aldrich, St. Louis, MO, USA) was used as an internal standard (IS). The standard and IS were diluted with acetonitrile and stored at −20°C. Acetonitrile, acetic acid (LC-MS grade), and ammonium acetate (JIS Special Grade) were purchased from Wako Pure Chemical. Purified water was prepared by MilliQ filtration (Millipore, Billerica, MA, USA). The brain levels of ACE were analyzed using previously reported analytical methods with some modifications (Seccia et al., 2008; Xiao et al., 2011; Zhang et al., 2012; Ueyama et al., 2014; Gbylik-Sikorska et al., 2015). A whole brain (0.34–0.49 g) was transferred to a 15 mL polypropylene (PP) tube and mixed with 500 μL of purified water and 50 μL of 50 ng/mL IS solution. The solution was homogenized using a Handy Ultrasonic Homogenizer (Microtec, Funabashi, Chiba, Japan) for 2 min. After adding 2 mL of acetonitrile, the sample solution was vortexed and ultrasonically extracted for 5 min. The extract was centrifuged at 3500 rpm for 5 min (Centrifugator, H-36, Kokusan, Saitama, Japan,), transferred to another 15 mL PP tube, and the solvent was evaporated with a centrifugal evaporator (CentriVap, Asahi Life Science, Saitama, Japan) for 90 min. The residue was re-dissolved in 0.1 mL of acetonitrile and diluted with purified water, up to 1 mL. The sample solution was passed through an Oasis HLB cartridge (1 mL/30 mg, Waters) that was pre-conditioned with 1 mL of acetonitrile and 1 mL of purified water. After rinsing with 1 mL of purified water, the ACE was eluted with 1 mL of acetonitrile/purified water (1:1, v/v). The eluates were evaporated with a centrifugal evaporator for 60 min and re-dissolved in 0.5 mL of acetonitrile/purified water (1:9, v/v) prior to the analysis. Quantification was performed using an ultra-high performance liquid chromatography (Nexera UHPLC, Shimadzu, Kyoto, Japan) coupled to a tandem mass spectrometry (LCMS8050, Shimadzu). The analytical column was Kinetex C18 (100 × 2.1 mm, 2.6 μm, Phenomenex, Torrance, CA, USA), and the injection volume was 10 μL. The mobile phase was (a) 17 mmol/L acetic acid and 5 mmol ammonium acetate in acetonitrile and (b) 17 mmol/L acetic acid and 5 mmol ammonium acetate in purified water; the flow rate was maintained at 0.4 mL/min. The gradient parameters were as follows: the initial condition (phase ratio a:b = 10:90) for 5 min, 40:60 for 5.5 min, 100:0 for 1 min, the initial condition for 6 min. A multiple reaction monitoring (MRM; precursor ion: m/z = 222.7 and product ion: m/z = 126.0) transition was used for the ACE quantification, and another MRM transition (precursor ion: m/z = 222.7 and product ion: m/z = 56.2) was used for confirmation. Quantification was performed using the relative response to IS. The linearity of the calibration curve of the ACE standard solution was confirmed from 0.1 to 100 ng/mL, with *r*^2^ > 0.998. The instrumental detection limit (IDL) of ACE was 0.024 ng/mL, which was calculated from the results of 7 replicate analyses of the standard solution. To calculate the method detection limit (MDL), 7 replicated analyses of a fortified blank sample were performed. The blank sample (0.4 mL of purified water) was spiked with 0.05 mL of 0.1 ng/mL standard solution and processed throughout the analytical procedure. The MDL was calculated to be 0.032 ng/g. The IS recovery during the sample analysis ranged from 63 to 98%. The extraction efficiency was evaluated by repeated extraction; the detected ACE level were below the IDL in the second and third extractions.

### General test procedure

When the offspring were 9–12 weeks old, 1 or 2 mice of both sexes were randomly selected from each litter for a behavioral test battery for socio-sexual and anxiety-related behaviors, consisting of tests for male and female sexual behaviors, aggressive behaviors, and the light-dark transition (LDT) test. The behavioral tests were performed during the dark phase (starting more than 2 h after lights off) of the light/dark cycle under red light. After completing the behavioral tests, the mice were sacrificed, and blood and brain samples were collected for the enzyme immunoassays and immunohistochemistry.

**Figure 1:**
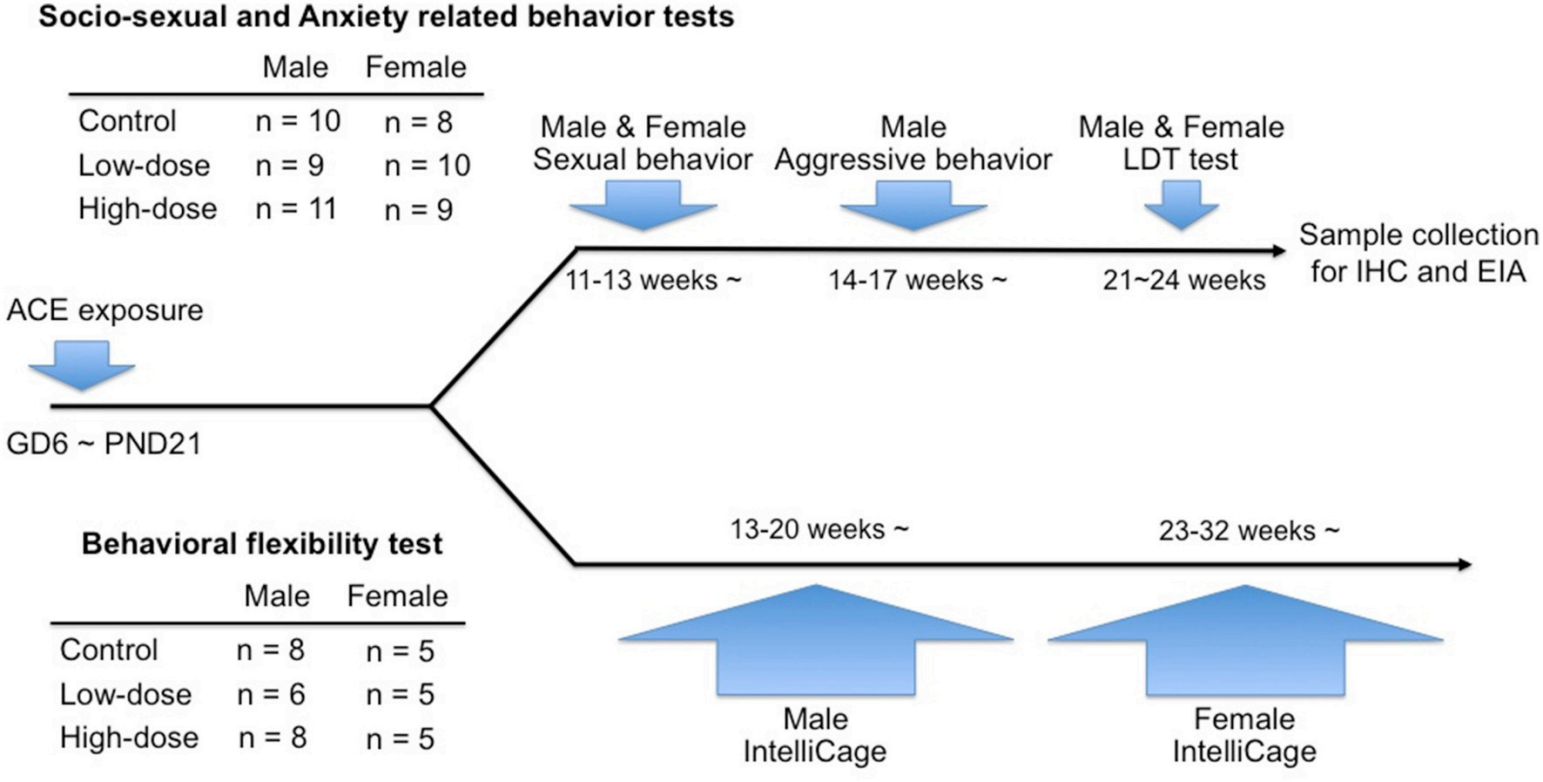
**Timeline of the behavioral tests in the study and information of the numbers of animals used in each cohort**.

Another 1–2 mice of both sexes from each litter (males: 13–20 weeks of age; females: 23–32 weeks of age) were assigned to the behavioral flexibility test using the IntelliCage apparatus (NewBehavior AG, Zurich, Switzerland). The experimental design for the tests and the number of animals for each group are shown in Figure 1.

### Male sexual behavior

The male mice were separated from their littermates and individually housed in plastic cages (5 × 22 × 12 cm). Starting 12–14 days later, each mouse was tested in its home cage for sexual behavior toward ovariectomized and sex-hormone-treated C57BL/6J female mice once weekly for 3 weeks. The duration of each trial was 30 min. All female stimulus mice were primed with subcutaneous injections of estradiol benzoate (EB) in sesame oil (10 μg/0.1 mL) twice before testing (at 24 and 48 h). The mice were also administered progesterone (P) in sesame oil (500 μg/0.1 mL) 4−6 h before testing to ensure high sexual receptivity. All tests were video-recorded; the numbers of attempted mounts, successful mounts, intromissions, and ejaculations were scored for each mouse.

### Male aggressive behavior

Five to Seven days after the last sexual behavior test, each male mouse was tested for its aggressive behavior against a gonadally intact, olfactory-bulbectomized C57BL/6J male intruder mouse using a resident-intruder paradigm. This test was performed weekly for 3 consecutive days, for a total of 9 trials. The duration of each trial was 15 min. All tests were video-recorded; the duration and number of aggressive bouts toward the intruder were scored for each mouse. The data for the 3 trials obtained each week were averaged for each mouse and used for statistical analysis. An aggressive bout was defined as a set of behavioral interactions that included at least one of the following behavioral actions toward the intruder: chasing, boxing, wrestling, biting, tail rattling, and offensive lateral attack. If the interval between 2 aggressive bouts did not exceed 3 s, the 2 bouts were considered to be continuous and scored as 1 bout.

### Female sexual behavior

At 10–12 weeks of age, the female mice were separated from their littermates and housed individually in plastic cages (5 × 22 × 12 cm). Fourteen to Sixteen days after the isolation, all mice were ovariectomized under isoflurane inhalation anesthesia. Fourteen to Sixteen days after the ovariectomy, each female mouse was tested for sexual behavior toward a sexually experienced ICR/JCL male mouse (CLEA Japan, Tokyo, Japan) in the male's home cage. This test was performed weekly, for a total of 3 trials. The female mice were subcutaneously injected with EB (5 μg/0.1 mL dissolved in sesame oil) at 24 and 48 h before testing, and P (250 μg/0.1 mL dissolved in sesame oil) at 4–6 h before testing. Each test lasted until females received 15 mounts or intromissions. The number of lordosis responses to the male mounts or intromissions was scored for each mouse. A lordosis quotient was calculated by dividing the number of lordosis responses by 15 mounts or intromissions (Ogawa et al., 1999).

### The LDT test

Each mouse was tested once for its emotional behaviors in the LDT apparatus for 10 min. The test apparatus consisted of enclosed dark and open-top light compartments (30 × 30 × 30 cm each) connected by an inner door way (3 × 3 cm) located in the center of the partition at the floor level. The open-top light compartment was brightly illuminated with a white light (350 lux). The latency to enter the light compartment, the cumulative time spent in the light compartment, and the total distance traveled in the light compartment were measured by an automated video tracking system (ANY-maze, Stoelting, USA). The data from 2 male mice (1 in the control group and 1 in the low-dose group) were excluded from the analysis because of recording errors.

### Sample collection

After completing the behavioral tests, mice were deeply anesthetized with sodium pentobarbital (60 mg/kg), and blood was collected from the left ventricle of each mouse. The mice were subjected to transcardial perfusion with 0.1 M phosphate-buffered saline (PBS; pH 7.2), followed by 4% paraformaldehyde (PFA) in 0.1 M PBS. Brains were removed, post-fixed overnight at 4°C with 4% PFA in 0.1 M PBS, and cryoprotected in 0.1M PBS containing 30% sucrose.

### Enzyme immunoassay for plasma testosterone

Samples were extracted from plasma (100 μl) with ethyl acetate, and testosterone concentrations were determined using a testosterone enzyme immunoassay kit (Cayman Chemicals, Ann Arbor, MI, USA), according to the manufacturer's instructions. All male samples and randomly selected samples from females (5 for each treatment group) subjected to the socio-sexual and anxiety behavior tests were analyzed.

### Immunohistochemistry

The brain samples were coronally sectioned at 30 μm thickness with 90 μm intervals on a freezing microtome. Sections were incubated in PBS-X (0.1 M PBS, pH 7.2 and 0.2% Triton X-100), containing 0.5% hydrogen peroxide for 20 min to inhibit the endogenous peroxidase activity, and then blocked in an incubation buffer (1% casein in PBS-X) for 2 h at room temperature. Tissue sections were then incubated with a rabbit polyclonal anti-vasopressin antiserum (1:4000; Immunostar Cat. #20069, Hudson, NY, USA) in incubation buffer for 24 h at 4°C. After the completion of the incubation process, the staining was visualized using the DAKO EnVision™ Detection System (Peroxidase/DAB+, K5007). Three anatomically matched sections (30 μm thickness, 90 μm intervals) containing the paraventricular nucleus (PVN, bregma 0.70–0.94 mm) were selected for each mouse. PVN images were photographed at a 10x magnification with a digital camera mounted on a light microscope (Leica DFC290 HD; Leica Microsystems, Wetzlar, Hesse, Germany). The total number of immunoreactive cells was bilaterally counted for each animal. Because of technical issues with the sample preservation and tissue preparation, only the number of samples denoted in **Figure 7** were used for the analysis.

### Evaluation of behavioral flexibility using the IntelliCage

Male and female mice were tested separately for their behavioral flexibility using the IntelliCage, a fully automated testing apparatus consisting of a large plastic cage (55 × 37.5 × 20.5 cm) equipped with 4 corner chambers (15 × 15 × 21 cm each). Male mice at the age of 13–20 weeks were introduced to the IntelliCage apparatus and housed for 57 days. The female mice were housed in the apparatus for 56 days, starting at the age of 23–32 weeks. The difference in test timing of test was due to the limited capacity of the IntelliCage. Two to Three days before being introduced to the apparatus, the mice were anesthetized with isoflurane and subcutaneously implanted a glass-covered transponder having a unique ID code for radiofrequency identification (Datamars, Temple, TX, USA).

The behavioral flexibility test paradigm was composed of an acquisition phase and serial reversal phases. Prior to the behavioral flexibility test, the mice were allowed to acclimatize to the IntelliCage for 9 days. In the acquisition phase after acclimatization, mice were allowed to learn the two rewarded corners and shuttle between them. Subsequently, the mice were subjected to serial reversal tasks, in which the diagonal spatial patterns of the rewarded corners was repetitively reversed every 4–7 sessions. In total, there were 57 sessions for the male mice and 56 sessions for the female mice, including the first 14 sessions of the acquisition phase. Additional sessions were conducted with 10 serial reversals for the male mice and 9 serial reversals for the female mice. The percentage of visits to the non-rewarded corners within the first 100 visits was defined as the discrimination error rate and used to analyze the inter-session comparisons of learning performance. Additionally, the nose-poke frequency per visit within the first 100 visits was calculated for each mouse as an index of compulsive repetitive behavior. The IntelliCage apparatus and behavioral flexibility test paradigm are described in details elsewhere (Endo et al., 2011, 2012). The data from session 38–41 (6th reversal phase) and session 46–53 (8th and 9th reversal phases) in the male mice were excluded from analysis because of a mechanical malfunction of the IntelliCage apparatus. Thus, in the **Figure 8A**, the sessions 42–45, which were the 7th reversal phase, are denoted as Rev 6 and sessions 54–57, which were the 10th reversal phase, are denoted as Rev 7.

### Statistical analyses

All data are presented as mean ± standard error of the mean (SEM). All data, except the comparison of the numbers of ejaculating males, were analyzed using an ANOVA, followed by a Fisher's PLSD *post hoc* test. The incidence of ejaculation during the male sexual behavior test was compared using χ^2^ tests. The differences were considered statistically significant when *P* < 0.05. All data were analyzed using the SPSS 19.0 statistical package (SPSS Inc., Chicago, IL, USA) or R software (The R Foundation for Statistical Computing, Vienna, Austria).

## Results

### Body weight/litter

In the male and female mice, no differences were found in BW/litter among the groups at birth, PND 21, or 23–26 weeks of age (Table 1).

**Table 1:** **Body weight per litter (BW/litter) at birth, weaning (PND21), and 23–26 weeks of age**.

	**Groups**	**Birth (g)**	**PND21 (g)**	**23–26 weeks (g)**
Male	Control	1.27 ± 0.02 (7)	7.85 ± 0.12 (6)	28.28 ± 0.57 (5)
	Low	1.30 ± 0.02 (6)	8.01 ± 0.37 (6)	30.09 ± 0.68 (5)
	High	1.31 ± 0.04 (9)	7.72 ± 0.39 (9)	28.08 ± 0.66 (6)
Female	Control	1.22 ± 0.02 (7)	7.38 ± 0.14 (6)	25.63 ± 0.72 (4)
	Low	1.29 ± 0.03 (6)	7.76 ± 0.27 (5)	25.82 ± 0.50 (5)
	High	1.31 ± 0.03 (9)	7.43 ± 0.31 (9)	24.85 ± 0.33 (5)

Data presented as mean ± SEM.

Number of dam is given in parentheses.

### Brain weight

No differences were found in the brain weights of either male or female mice at PND 21 (Table 2). There were no differences in the brain-to-body weight ratio either [data not shown].

**Table 2:** **Brain weight at PND 21**.

	**Groups**	**Brain weight (g)**
Male	Control (*n* = 6)	0.41 ± 0.007
	Low (*n* = 2)	0.44
	High (*n* = 4)	0.41 ± 0.008
Female	Control (*n* = 6)	0.40 ± 0.003
	Low (*n* = 4)	0.41 ± 0.003
	High (*n* = 6)	0.39 ± 0.013

Data presented as mean ± SEM.

### Brain residual concentration analysis

The brain residual concentration of ACE was measured in 6 mice (3 male and 3 female) from the control group and 6 mice (3 male and 3 female) from the high-dose group. The concentrations in the high-dose group were 1.29 ± 0.46 and 1.23 ± 0.20 ng/g in males and females, respectively (Table 3). In contrast, the concentrations were below the MDL in the control group for both sexes.

**Table 3:** **Residual concentration of ACE in the brains of offspring at PND 21**.

	**Groups**	**Brain residual concentration (ng/mg tissue)**
Male	Control (*n* = 3)	< MDL
	High dose group (*n* = 3)	1.29 ± 0.46
Female	Control (*n* = 3)	< MDL
	High dose group (*n* = 3)	1.23 ± 0.20

MDL: The method detection limit.

Data presented as mean ± SEM.

### Male sexual behavior

**Figure 2:**
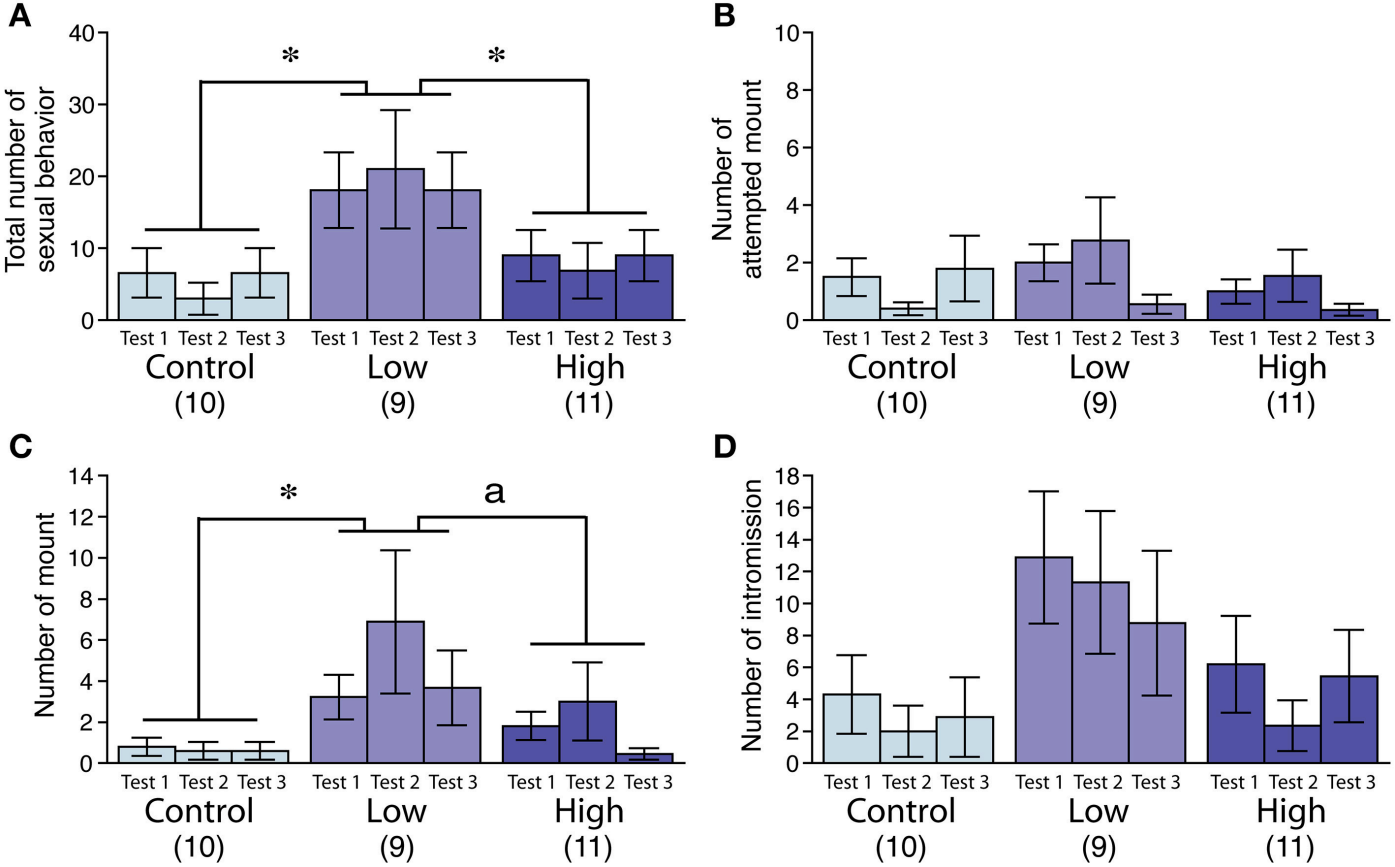
**Effects of developmental ACE exposure on male sexual behavior**. Comparison of **(A)** the total number of sexual behaviors, as well as the numbers for each male sexual behavior component, including **(B)** attempted mounts, **(C)** mounts, and **(D)** intromissions across the treatment groups. The numbers of animals used are indicated in parentheses. The data are presented as the mean ± SEM. ^*^P < 0.05, ^a^*P* < 0.1.

In the male sexual behavior test (Figures 2A–D), the total number of sexual behaviors was significantly increased in the low-dose group [*F*_(2, 27)_ = 3.72, *P* < 0.05; Fisher's PLSD, *P* < 0.05, low-dose group vs. control and high-dose groups; Figure 2A], particularly for the mean number of mounts [*F*_(2, 27)_ = 3.77, *P* < 0.05; Fisher's PLSD, *P* < 0.05, low-dose group vs. control group; Figure 2C]. We found no significant difference in the incidence of ejaculation during the tests (Table 4).

**Table 4:** **Number of male mice demonstrated ejaculation in each test**.

**Groups**	**Test 1**	**Test 2**	**Test 3**
Control	2/10 (20%)	2/10 (20%)	2/10 (20%)
Low	3/9 (33.3%)	2/9 (22.2%)	5/9 (55.6%)
High	2/11 (18.2%)	2/11 (18.2%)	4/11 (36.4%)

Number of mice ejaculated/total number of mice tested; the percentage is given in parentheses.

### Male aggressive behavior

**Figure 3:**
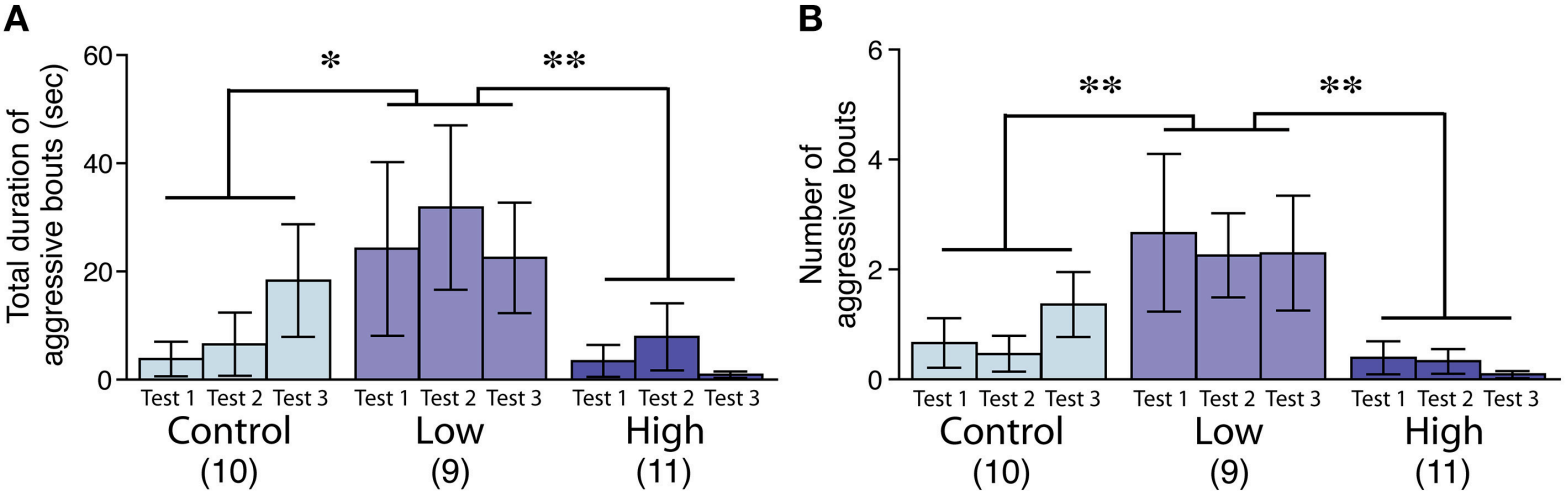
**Effects of developmental ACE exposure on male aggressive behavior. (A)** The total duration and **(B)** the number of aggressive bouts toward the intruder stimuli. The numbers of animals used are indicated in parentheses. The data are presented as the mean ± SEM. ^**^P < 0.01, ^*^P < 0.05.

The aggression level in the low-dose group was significantly increased compared to that of the control and high-dose groups, as measured by the total duration [*F*_(2, 27)_ = 4.44, *P* < 0.05; Fisher's PLSD, *P* < 0.05, low-dose group vs. control; *P* < 0.01, low-dose group vs. high-dose group; Figure 3A] and the number of bouts [*F*_(2, 27)_ = 6.24, *P* < 0.01; Fisher's PLSD, *P* < 0.01, low-dose group vs. control and high-dose groups; Figure 3B].

### Female sexual behavior

**Figure 4:**
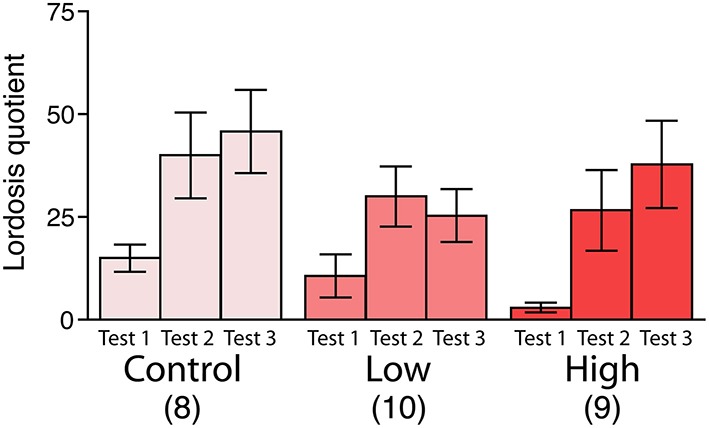
**Effects of developmental ACE exposure on female sexual behavior**. The comparison of the lordosis quotient [(the number of lordosis responses/15 mounts or intromissions) ^*^ 100]. The numbers of animals used are indicated in parentheses. The data are presented as the mean ± SEM.

No significant differences were found in the lordosis quotient among the groups, whereas the lordosis quotient increased with repeated testing in all groups [*F*_(2, 48)_ = 12.83, *P* < 0.001; Figure 4].

### The LDT test

**Figure 5:**
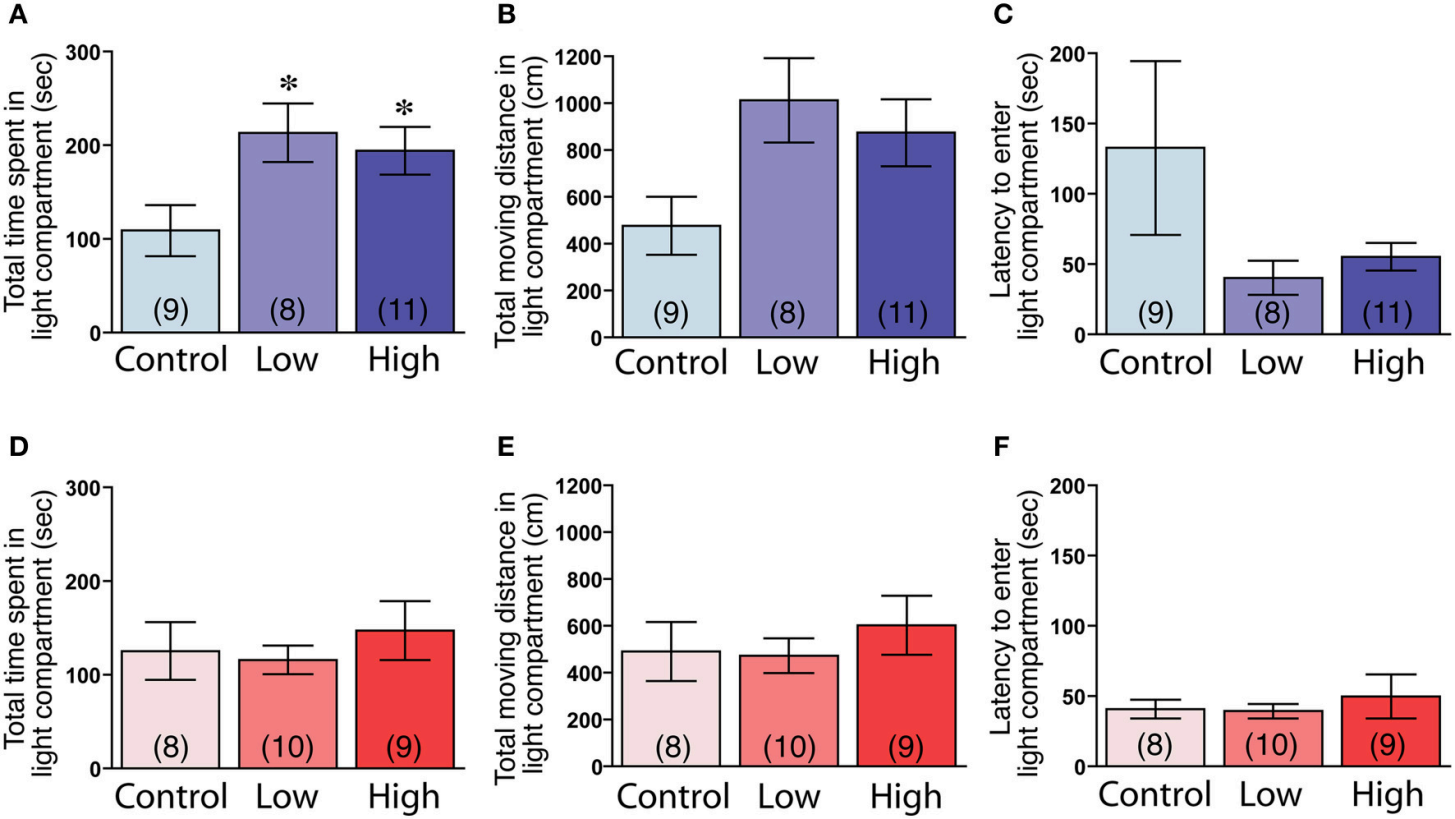
**Effects of developmental ACE exposure on anxiety-related behaviors, as measured in the light-dark transition test. (A–C)** Male and **(D–F)** female mice of each treatment group. The comparison of **(A,D)** the total time spent in, **(B,E)** the total moving distance in, and **(C,F)** the latency to enter the light compartment of the light-dark transition apparatus. The numbers of animals used are indicated in parentheses. The data are presented as the mean ± SEM. ^*^P < 0.05 vs. control.

The male mice in both the low-dose and high-dose groups spent significantly more time in the light compartment compared to the control group [*F*_(2, 25)_ = 3.83, *P* < 0.05; Fisher's PLSD, *P* < 0.05, low-dose and high-dose groups vs. control group; Figure 5A]. The male mice in the low-dose and high-dose groups tended to travel longer distances in the light compartment compared to the control group [*F*_(2, 25)_ = 3.27, *P* = 0.055; Figure 5B]. We found no significant differences in the latency to enter the light compartment (Figure 5C). In contrast to the males, there were no significant group differences in the females in the time spent in the light compartment (Figure 5D), the total distance traveled in the light compartment (Figure 5E) and the latency to enter the light compartment (Figure 5F).

### Plasma testosterone levels

**Figure 6:**
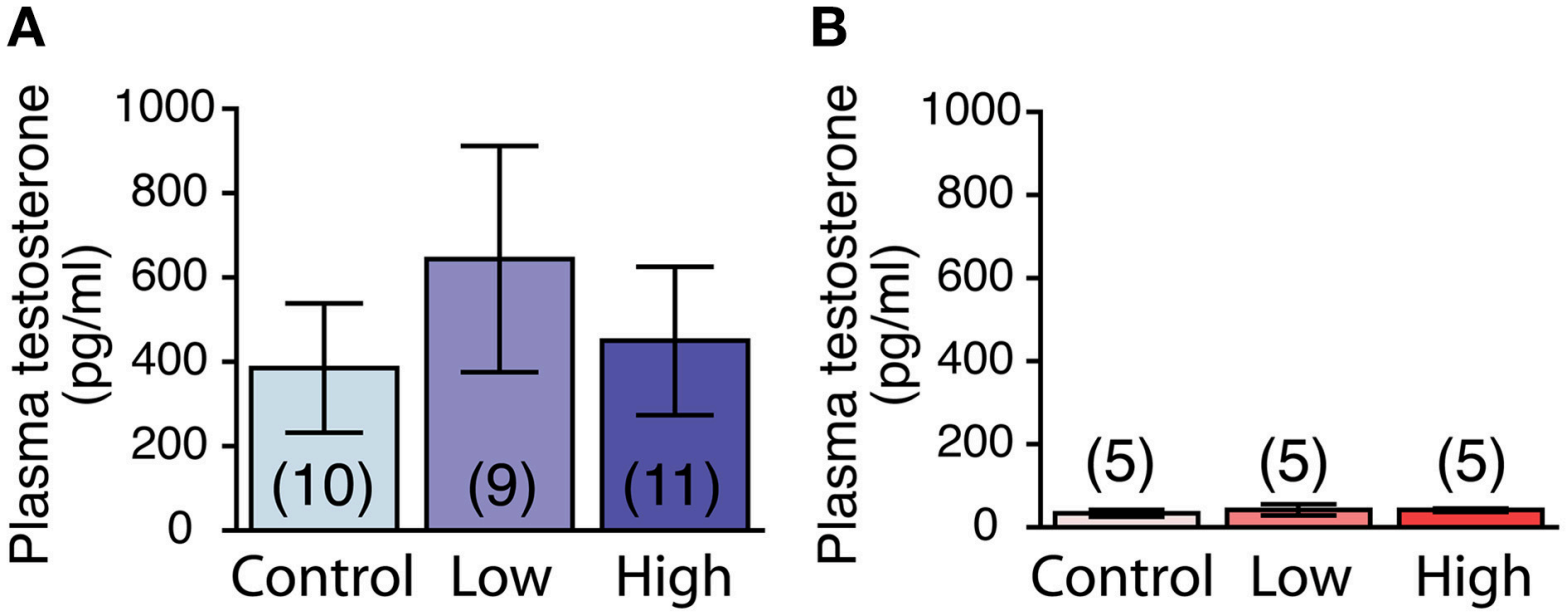
**Effects of developmental ACE exposure on plasma testosterone levels. (A)** Male and **(B)** female mice of each treatment group. The numbers of animals used are indicated in parentheses. The data are presented as the mean ± SEM.

The plasma testosterone levels were significantly higher in the males compared to those of the females, regardless of treatment [*F*_(1, 39)_ = 7.48, *P* < 0.01; Figures 6A,B]. No main effect of ACE exposure was found on the plasma testosterone levels in either the males (Figure 6A) or females (Figure 6B).

### AVP immunoreactivity in the PVN

**Figure 7:**
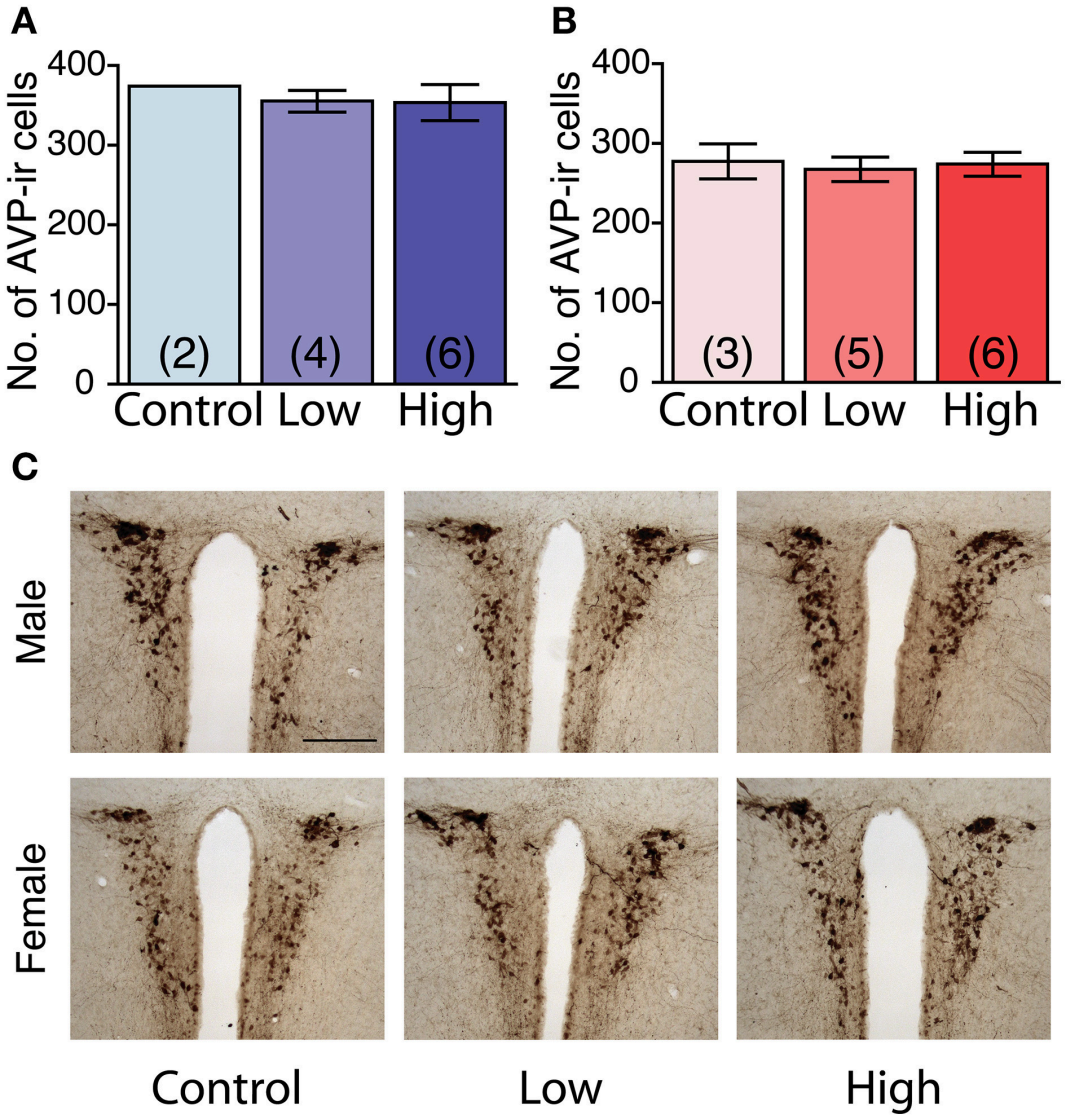
**Effects of developmental ACE exposure on the number of vasopressin (AVP) immunoreactive (ir) cells in the paraventricular nucleus (PVN). (A)** Male and **(B)** female mice of each treatment group. The data are presented as the mean ± SEM. **(C)** Representative photomicrographs of AVP-ir cells in brain sections from males (top panels) and females (bottom panels) of each treatment group. The numbers of animals used are indicated in parentheses **(A,B)**. The scale bar represents 200 μm.

No main effect of ACE exposure was found on the number of AVP immunoreactive cells in either the males (Figure 7A) or females (Figure 7B). The number of AVP-immunoreactive cells was significantly higher in the males than in the females, regardless of treatment [*F*_(1, 20)_ = 26.7, *P* < 0.01; Figures 7A,B] as shown in representative photomicrographs (Figure 7C).

### Behavioral flexibility test

**Figure 8:**
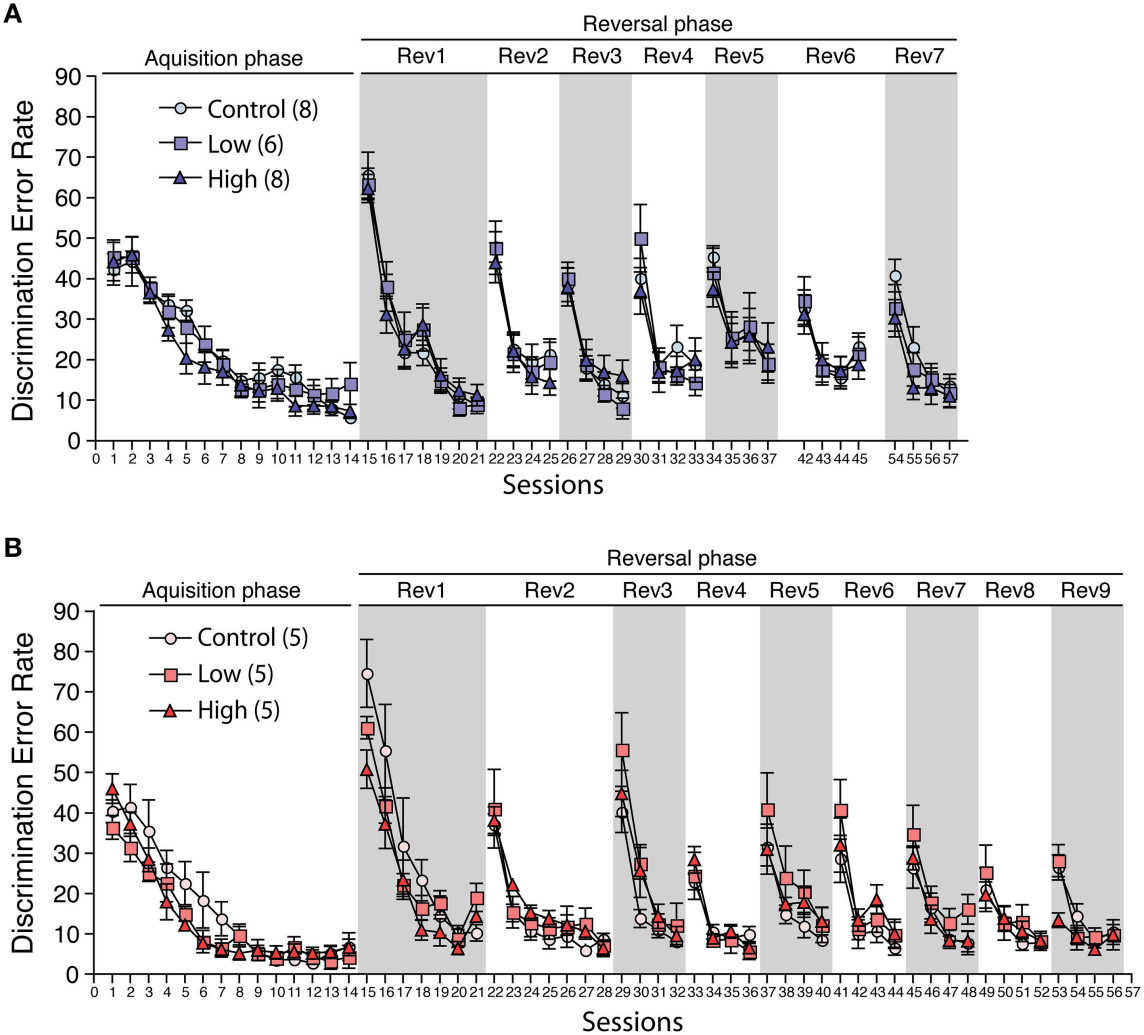
**Effects of developmental ACE exposure on behavioral flexibility**. The comparison of the discrimination error rates (the number of discrimination errors in the first 100 corner visits in the session) in **(A)** male and **(B)** female mice. The numbers of animals used are indicated in parentheses. The data are presented as the mean ± SEM. The data for session 38–41 (6th reversal phase) and session 46–53 (8th and 9th reversal phases) in male were omitted due to the technical issues with the IntelliCage apparatus. Thus, the session 42 to 45 is denoted as Rev 6 and the session 54–57 is denoted as Rev 7 on **(A)**.

During the acclimatization period, the number of corner visits per week in the IntelliCage did not differ between the groups in both males and females (Supplementary Figure 1), indicating the possibility that the developmental exposure to ACE does not affect home cage activity. In the acquisition phase, the male and female mice in all groups showed a decrease in the discrimination error rate with repeated sessions. No statistical differences were found between the groups, indicating that all groups could acquire the first task (Figures 8A,B). In the serial reversal phase, the male and female mice in all groups showed a similar pattern in the discrimination error rate in the reversal stage (Rev), indicating that the developmental ACE exposure had no effect on the spatial learning ability and behavioral flexibility during adulthood. We measured the nose-poke/visit ratio, which is an index of behavioral impulsivity, against rewards in the home cage, but found no significant group differences.

## Discussion

We evaluated the general physiological parameters, such as body and brain weights, during the developmental period of mice exposed to ACE *in utero* and via lactation, as well as a battery of socio-sexual and anxiety-related behaviors during their adulthood. ACE was found in the brains of pups of the high-dose group at PND 21, using a residual concentration analysis. These data showed that absorbed ACE was transferred into the developing brain. The ACE exposure at the used doses did not alter the body and brain weights. In addition, no impairments in behavioral flexibility were found in the *in utero* and lactational ACE-exposed adult mice. On the other hand, *in utero* and lactational ACE exposure altered socio-sexual and anxiety-related behaviors in males.

Although, the binding affinity of ACE to nAChRs is approximately 70–80 times lower than that of nicotine (Tomizawa and Casida, 1999; Picciotto et al., 2001), ACE can be bound to nAChRs containing α_4_ and β_2_ subunits, which are known to mediate the effects of nicotine. Based on this binding affinity difference, we propose that the effect of ACE on certain behaviors may correspond to the effect of nicotine at lower doses. Therefore, in our discussion below, we attempt to compare the present results to previous reports demonstrating the effects of developmental nicotine exposure at relatively low doses, when possible.

We found significant changes in the socio-sexual and anxiety-related behaviors in male mice exposed to ACE *in utero* and via lactation. The most significant change was found by the LDT test. ACE exposure at low or high doses prolonged the time spent in the light compartment, indicating reduction of anxiety. This result suggests that there may be altered emotional responses in the ACE-exposed male mice. There have been many reports regarding the effects of nicotine exposure on anxiety-related behaviors during the developmental period. However, the effects of perinatal nicotine exposure reported in these studies are bidirectional, positive or negative, depending on the experimental conditions such as the dose, time of administration, and the behavioral test paradigm. Maternal nicotine exposure at a dose of 0.5 mg/kg BW from GD 10 to delivery in mice is reported to decrease anxiety-related parameters measured in the elevated plus-maze test in male offspring (Ajarem and Ahmad, 1998). In contrast, male mice whose mothers were exposed to nicotine (0.2 mg/ml) in drinking water throughout gestation or from GD 14 to delivery have been reported to exhibit increased anxiety-related responses in both the elevated plus-maze and LDT tests (Alkam et al., 2013). Vaglenova et al. (2004) reported that nicotine exposure in pregnant rats at a daily dose of 6.0 mg/kg BW from GD 3 to delivery also causes increased anxiety-related responses in their offspring as assessed in the elevated plus-maze test. The relationship between developmental nicotine exposure and anxiety shown by many studies allows us to speculate that the neural circuitry responsible for anxiety is vulnerable to cholinergic agents, although reservation is warranted because of inconsistencies among previous studies. In addition, perinatal nicotine exposure is reported to cause hyperactivity in rodents (Ajarem and Ahmad, 1998; Thomas et al., 2000; Pauly et al., 2004; Vaglenova et al., 2004; Paz et al., 2007; Heath et al., 2010; Alkam et al., 2013). We did not find any differences between the groups in corner visits in the IntelliCage, demonstrating no differences in home cage activity between the groups. However, mice exposed to ACE at both the low and high doses tended to travel longer distances in the light compartment, suggesting the possibility that ACE exposure may have induced hyperactivity under specific stressful conditions. Therefore, the longer times spent in the light compartment in the LDT test may reflect not only decreased anxiety, but also hyperactivity under specific stressful conditions. Future experiments measuring locomotor activity with or without stress are necessary to clarify whether ACE exposure decreases low anxiety, induces hyperactivity, or both under stressful conditions.

On socio-sexual behaviors, the effect of ACE appeared to be dose-specific. ACE exposure at the low dose, but not at the high dose, significantly increased male sexual and aggressive behaviors. Socio-sexual behaviors are governed by neural circuits involving various brain regions (Newman, 1999). Peripheral sex steroid hormones play an essential role in the formation of these neural circuits during the critical period. Generally in mammals, the critical period can extend from late gestational period to the early life (Hull and Dominguez, 2007; Nelson and Trainor, 2007). The cholinergic system is also thought to play a role in the formation of the neural bases of these behaviors (Dwyer et al., 2009; Blood-Siegfried and Rende, 2010). Any agents that block or enhance acetylcholine transmission may interfere with this formation.

The idea that low-dose chemical exposure alters specific behaviors has been proposed in previous studies. Dioxin and bisphenol A induce their toxicities in a non-monotonic manner (Endo et al., 2012; Vandenberg, 2014). The actions of nicotine also appear to be complex and non-monotonic, especially when given *in vivo* (Anderson and Brunzell, 2012, 2015; Alkam et al., 2013; Abreu-Villaça et al., 2015). Behaviors as well as specific neural circuit formation may potentially be dysregulated by nicotine in a non-monotonic manner. For example, the effect of developmental nicotine exposure on the development of the vasopressinergic system is complex. Prenatal nicotine exposure at a relatively high dose (6 mg/kg/day throughout the gestational period) greatly reduced vasopressin production and release in the hypothalamus of male rats (Zbuzek and Zbuzek, 1992). Although, the species difference must be taken into consideration, prenatal nicotine exposure at a relatively low dose (total amount of 1.05 mg throughout the gestational period) has been reported to increase the number of vasopressin cell bodies and fibers in the hypothalamus of the golden hamster (Rossi et al., 2003). Since vasopressinergic systems regulate both sexual and aggressive behaviors in a facilitative manner (Ho et al., 2010; Stevenson and Caldwell, 2012), we speculated that ACE exposure specifically at the low dose enhanced the development of neural networks involved in vasopressinergic systems. However, we did not find any differences in the number of AVP-immunoreactive cells in the PVN. Therefore, it is unlikely that *in utero* and lactational ACE exposure at the low dose increased aggression and sexuality in adulthood through alterations of the vasopressinergic system in the PVN. We could not examine the numbers of AVP-immunoreactive cells in areas outside the PVN, such as the bed nucleus of the stria terminalis (BNST), a region known to be critically involved in the facilitation of male socio-sexual behaviors (Newman, 1999; Nelson and Trainor, 2007). Further studies are needed to clarify whether ACE affects AVP immunoreactive cells in brain regions other than the PVN.

There have been several reports regarding the effects of in utero nicotine exposure on plasma testosterone levels in animal models, but these reports are also inconsistent with each other, presumably due to differing exposure conditions. On one hand, the plasma testosterone levels decreased in 10-week-old male rat offspring born to dams that had been administered nicotine at a dose of 0.5 mg/kg throughout gestation (Segarra and Strand, 1989). In contrast, the plasma testosterone levels increased in 13-week-old male rat offspring born to dams that had been given nicotine at a dose of 2 mg/kg throughout gestation increased (Paccola et al., 2014). In our study, we did not find any differences in plasma testosterone levels measured at 23–26 weeks of age, which excludes the possibility that increased testosterone levels in adult males caused the abnormalities in the socio-sexual and emotional behaviors. However, we did not determine plasma testosterone levels during the perinatal period, which is a critical period for the testosterone-induced masculinization of socio-sexual behaviors. Therefore, we cannot exclude the possibility that developmental ACE exposure altered the testosterone levels during the perinatal periods, resulting in the impaired sexual differentiation of socio-sexual behaviors.

Behavioral flexibility was unaffected by developmental ACE exposure under our exposure conditions. Behavioral flexibility is considered a part of executive function, which is dependent on hippocampal and prefrontal cortical function (Kosaki and Watanabe, 2012; Malá et al., 2015). Various studies have reported the negative effects of developmental nicotine exposure at relatively high doses on the cognitive performance of offspring (Yanai et al., 1992; Wickström, 2007; Parameshwaran et al., 2012). However, the effects of developmental nicotine exposure to neonicotinoids or lower doses of nicotine have not been clarified for cognitive functions. Our study demonstrated that the developmental ACE exposure did not affect the cognitive functions, at least at the ACE doses and animal ages tested in this study. However, we cannot exclude the possibility that late-onset impairment of cognitive function occurs in mice exposed to ACE. Since malfunction of the cholinergic system is associated with cognitive deficits such as those in Alzheimer's disease (Schliebs and Arendt, 2011), examining behavioral flexibility in aged mice exposed to ACE perinatally would be an interesting avenue of our research.

Sex differences in the effects of ACE exposure on socio-sexual and emotional behaviors should also be addressed since *in utero* and lactational ACE exposure at either dose had no effect on female sexual behavior and anxiety. The present study was unable to elucidate the neuropathologies underlying the sex-specific effect of ACE. Several lines of evidence suggest that the male is more susceptible to developmental nicotine exposure than the female (Fung and Lau, 1989; Ribary and Lichtensteiger, 1989; von Ziegler et al., 1991; Pauly et al., 2004). The sex-specific effect of ACE observed in this study is consistent with the reported male–biased effects of nicotine, possibly suggesting that nicotine and ACE elicit toxic effects through a common sex-specific pathway.

It is also important to mention that ACE was administered to mice throughout the gestational and lactational periods in this study. The effect of developmental nicotine exposure has been reported to vary depending on the timing of exposure (Alkam et al., 2013). Therefore, if ACE shares a common toxic pathway with nicotine, it is highly likely that the effect of ACE also varies depending on the timing of exposure. The developmental action of ACE in different time-windows should be further examined in future studies.

## Conclusion

Our results suggest the possibility that ACE affects socio-sexual and anxiety-related behaviors in a male-specific manner. Further experiments are needed to understand the behavioral alterations and examine the mechanisms underlying the ACE-induced impairments in brain function. The action of ACE appears to be non-monotonic for the socio-sexual behaviors, as the effects were only found in mice exposed to ACE at low doses. Further empirical studies using mouse models are required to evaluate whether ACE doses equivalent to the human exposure level have detrimental effects.

## Author contributions

KS, TI, TW, SN, TK, GS, SH, and FM designed the experiment. KS, TI, JY, TW, MY, TK, and FM performed the experiment. KS, TI, JY, MY, and FM analyzed the data. KS wrote the manuscript. KN, CT, and FM critically revised the manuscript.

### Conflict of interest statement

The authors declare that the research was conducted in the absence of any commercial or financial relationships that could be construed as a potential conflict of interest.
