# Supplementary material for: In utero and Lactational Exposure to Acetamiprid Induces Abnormalities in Socio-Sexual and Anxiety-Related Behaviors of Male Mice
Source: Front Neurosci. 2016 Jun 3;10:228. doi: 10.3389/fnins.2016.00228 (PMC4891355; doi:10.3389/fnins.2016.00228)
Supplement: Supplementary file 1 [file DataSheet1.DOCX]

**Supplemetary Fig. 1⏐Number of corner visit during the acclimatization period. (A)** Male and **(B)** female mice of each treatment group. The numbers of animals used are indicated in parentheses. The data are presented as the mean ± SEM.
