# Supplementary figures and images for: In utero and Lactational Exposure to Acetamiprid Induces Abnormalities in Socio-Sexual and Anxiety-Related Behaviors of Male Mice
Source: Front Neurosci. 2016 Jun 3;10:228. doi: 10.3389/fnins.2016.00228 (PMC4891355; doi:10.3389/fnins.2016.00228)

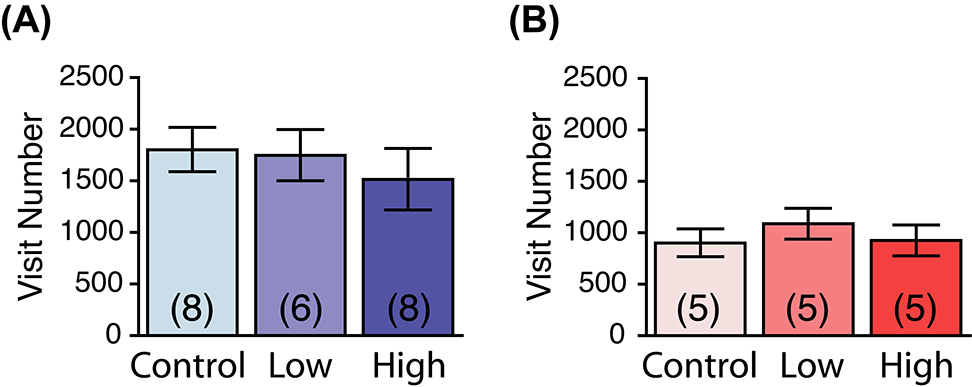

Supplement: Supplementary file 2 [file Image1.TIF]
